# Fronto-parietal single-trial brain connectivity benefits successful memory recognition

**DOI:** 10.1515/tnsci-2022-0265

**Published:** 2022-12-31

**Authors:** Soyeon Jun, Yihyun Joo, Youjin Sim, Chuyun Pyo, Keunsoo Ham

**Affiliations:** Neuroscience Research Institute, Seoul National University College of Medicine, Seoul, South Korea; National Forensic Services, Forensic Medical Examination Division, 10, Ipchun-ro, Wonju-si, Gangwon-do, 26460, South Korea

**Keywords:** memory, recognition, theta, brain connectivity, EEG, fronto-parietal, mutual information

## Abstract

Successful recognition has been known to produce distinct patterns of neural activity. Many studies have used spectral power or event-related potentials of single recognition-specific regions as classification features. However, this does not accurately reflect the mechanisms behind recognition, in that recognition requires multiple brain regions to work together. Hence, classification accuracy of subsequent memory performance could be improved by using functional connectivity within memory-related brain networks instead of using local brain activity as classifiers. In this study, we examined electroencephalography (EEG) signals while performing a word recognition memory task. Recorded EEG signals were collected using a 32-channel cap. Connectivity measures related to the left hemispheric fronto-parietal connectivity (P3 and F3) were found to contribute to the accurate recognition of previously studied memory items. Classification of subsequent memory outcome using connectivity features revealed that the classifier with support vector machine achieved the highest classification accuracy of 86.79 ± 5.93% (mean ± standard deviation) by using theta (3–8 Hz) connectivity during successful recognition trials. The results strongly suggest that highly accurate classification of subsequent memory outcome can be achieved by using single-trial functional connectivity.

## 1 Introduction

Successful recognition can be defined as the accurate retrieval of previously encoded information. Researchers have been exploring the neural correlates of successful recognition, as well as ways in which these measures can be used to evaluate memory performance. Such attempts hold significance especially for the criminal justice system. In situations where physical evidence cannot be obtained, eyewitness testimonies serve a significant role in the criminal justice system. However, studies have shown that the testimonies of compliant witnesses without the intention of deception can be prone to error [1], and the consequences of inaccurate eyewitness testimonies can be dire. In fact, eyewitness misidentification is the most common contributing factor to wrongful convictions in the United States [2]. One solution to this inherent problem of inaccurate witness testimonies could be to devise a method to scientifically assess the accuracy of the eyewitness’s memory. Accurate predictions of the eyewitness’s memory performance with neural measures would greatly increase the justice system’s ability to discern which testimonies should be used as evidence.

Electroencephalography (EEG) has been incorporated in exploring the neural correlates of successful recognition. Previous studies have identified multiple event-related potential (ERP) components that show an “old/new effect,” a difference in ERP amplitude between the successful recognition of “old” (witnessed) and “new” (not witnessed) items [3,4]. An example of this would be the late positive complex (LPC), a positive-going ERP component traditionally observed in the parietal regions between 500 and 800 ms. The LPC amplitude evoked by correctly recognized old (hits) items is known to be larger compared to the LPC amplitude evoked by new (correct rejections, CR) test items. This is a left-lateralized phenomenon otherwise known as the parietal old–new effect [5,6], a neural correlate of successful recognition [7,8].

Studies using the parietal old–new effect as a classifier for machine learning algorithms have found the maximum accuracy of this approach to be 57.43% [4]. Along with ERP correlates, rhythmic oscillations in electrical charge are associated with local and global neural interaction and integration [9]. Specifically, previous research showed activity in the theta (3–8 Hz) and gamma (30–50 Hz) frequency bands to be related to successful recognition [10–13]. These studies have shown that it is plausible to incorporate neural measures in predicting one’s subsequent memory outcome.

Recent trends have been shifting toward taking more global, network-level approaches to classify various cognitive functions [14]. Previous EEG-based connectivity studies showed the relations between cognitive emotion regulation strategies and EEG synchronization levels during resting states [15]. In addition, discrete emotional states can be classified by EEG-based graph theoretical network measure [16]. Furthermore, Gupta et al. classified cognitive workload with EEG-based connectivity along with different deep learning algorithms and achieved a classification accuracy of 95.92%. A significant benefit of using brain connectivity measures as classifying features lies in the fact that connectivity measures take into consideration the communication between different brain regions during memory processing. Given the successful application of functional connectivity approaches in the context of memory performance [17], utilizing a network-level approach as such could render a more comprehensive picture of the neural bases of recognition [18]. Brain connectivity approach could be taken to successfully estimate recognition memory performance, even on a single-participant level. Thus, a network-based connectivity approach could be a better suited measure to accurately evaluate a witness’s memory performance.

For the brain connectivity approach, mutual information (MI) has been adopted for the connectivity analysis in this study. MI is a measure of the amount of dependency between two signals. Compared to linear correlation, MI is a universal measurement, since it could measure nonlinear dependency. The temporal series of mean frequency band signals were used to compute the cross-MI between regions of interest (ROIs). MI values between ROIs can be determined using the probability density function, as follows:

{\rm{MI}}={{\rm{MI}}}_{{XY}}={{\rm{MI}}}_{{YX}}={\rm{MI}}(X,Y)\hspace{1.5em}=\sum p(X,Y){\rm{\log }}\frac{p(X\left,Y)}{p\left(X)\left\bullet p\left(Y)},]

where *p*(*X*,*Y*) is the joint probability distribution function of variables *X* and *Y*, and *p*(*X*) and *p*(*Y*) are the marginal probability distribution functions of *X* and *Y*, respectively.

Despite the robust literature suggesting the feasibility of using frequency-specific functional connectivity to classify memory, not many studies to date have taken a machine learning approach in classifying subsequent memory outcome using single-trial functional connectivity. If this approach is successful in improving the accuracy of memory outcome prediction, it may be the key in improving the accuracy of EEG-based eyewitness-memory evaluation methods. Therefore, this study aims to explore the frequency-specific connectivity patterns during the successful recognition of witnessed and non-witnessed items. Moreover, we aim to evaluate whether using single-trial connectivity features as classifiers in a support vector machine (SVM) can yield significantly accurate results in classifying subsequent memory outcome.

## 2 Methods

### 2.1 Participants

A total of 40 healthy participants (19 females, 21 males, age = 23.27 years, age range: 19–29 years, SD = 2.60) were recruited by means of advertising. The subjects were rewarded with monetary compensation for their participation. Prior to the procedure, any participants who may have impaired vision was advised to use any visual aid necessary to ensure intact perceptual accuracy. Analysis was restricted to include only those (*n* = 30) participants whose data fulfilled the analysis criteria (Table 1).

**Table 1:** Subject demographics

Variable	*n* = 40	Mean	Standard deviation	%
Age	40	23.27	2.6	100.0
Gender				
Female	19			47.5
Male	21			52.5
Handedness				
Left	3			7.5
Right	37			92.5


**Ethical approval:** The research related to human use has been complied with all the relevant national regulations, institutional policies and in accordance the tenets of the Helsinki Declaration, and has been approved by Institutional Review Board of the National Forensic Service (906-200228-HR-001-01).
**Informed consent:** Informed consent has been obtained from all individuals included in this study.

### 2.2 Experimental design

**Figure 1:**
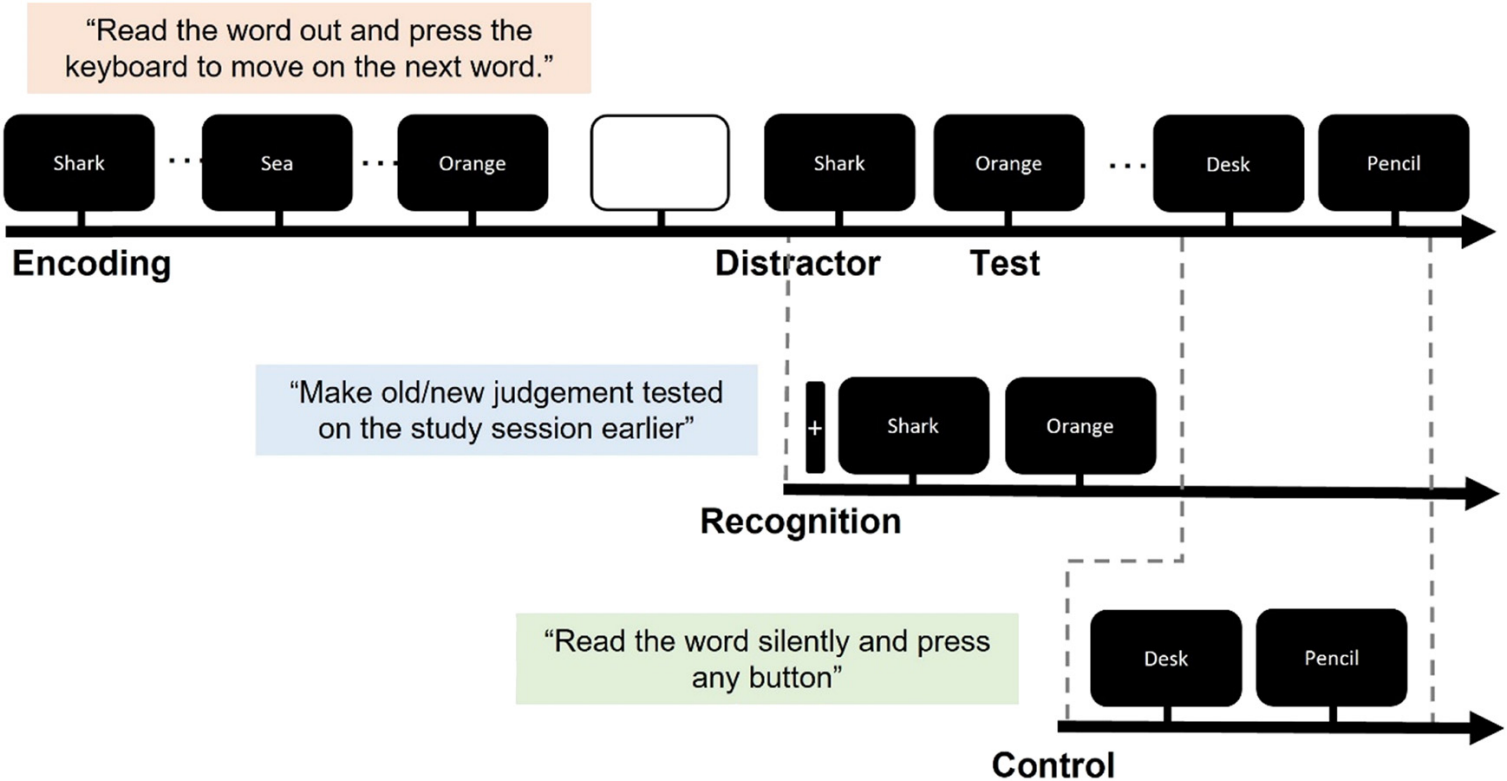
Example of the timeline of the word recognition memory task. The task was comprised of three successive stages of encoding, distractor, and test phase. The test phase consisted of ten recognition blocks and ten control blocks presented in a randomized order, with an interval of 5,000 ms in between each block. EEG data were collected during the retrieval phase only.

A diagram of the experimental paradigm is displayed in Figure 1. A modified old–new recognition task was used to explore the functional brain connectivity patterns during successful recognition of witnessed items (hit), successful recognition of not witnessed items (CR), and resting state (control). After obtaining informed consent, the participants completed an encoding task during which they studied a list of 120 Korean words for a subsequent memory test. The encoding task was programmed in E-Prime 2.0 (Psychology Software Tools, Inc.). During the encoding task, participants were instructed to read out the word loud that appeared on a 15 in. laptop screen and to press a button to proceed. The task was programmed so that each word appeared five times, without the same word appearing more than twice in a row.

The encoding task was followed by a recognition task after a 30 min delay. During the recognition task, the participants were situated in a comfortable chair 60 in. away from a 24 in. monitor. STIM2 (Compumedics, Neuroscan) was used to program the task and to record the behavioral responses. The recognition task consisted of ten recognition blocks and ten control blocks presented in a randomized order, with an interval of 5,000 ms in between each block. During the recognition blocks, participants viewed a fixation cross for 14–16 s, followed by the presentation of six previously studied words (old) and six novel words (new). Each word was presented on the screen for 2,800 ( \pm ] 200) ms. The participants were instructed to make a judgment whether they remember studying the word during the previous study session (old) or not (new). “Old” responses were made by pressing the left button with the left thumb, and “new” responses were made by pressing the right button with the right thumb. The control blocks followed the same temporal structure, except that the participants were presented with unstudied words (new) and were instructed to read the presented words silently without making recognition judgments, and to press any button. The control task was designed as such to physically mimic the recognition task (i.e., button pressing, visual/semantic processing of the presented words, etc.) without evoking recognition. The test session took on average 30 min to complete. EEG data were collected during the test phase only.

### 2.3 EEG equipment and data collection

EEG signals were obtained through the SynAmps2 System (Compumedics, Neuroscan) in an electrically shielded, sound-attenuated room. Recorded EEG signals were collected using a 32-channel Quick-Cap, which had 30 Ag/AgCl electrodes (FP1, FP2, F7, F3, FZ, F4, F8, FT7, FC3, FCZ, FC4, FT8, T7, C3, CZ, C4, T8, TP7, CP3, CZ, CP4, TP8, P7, P3, PZ, P4, P8, O1, O2, and OZ) mounted on it according to the international 10–20 system and two reference electrodes placed at the opposite lateral mastoids. EEG data were sampled at 500 Hz. To assure the collection of high-quality data, impedance for the previously mentioned channels was kept below 5 kΩ. Horizontal and vertical eye movements were monitored with electrooculogram (EOG) electrodes. Horizontal electrooculogram (hEOG) electrodes were placed above and below the left eye, and vertical electrooculogram (vEOG) electrodes were placed near the outer canthi of each eye. Impedance for the EOG channels was kept below 10 kΩ.

The EEG and EOG signals were baseline corrected and bandpass filtered between 0.3 and 30 Hz. Ocular artifacts were corrected by applying the covariance method with the data from both hEOG and vEOG channels. The data for each were then epoched from −200 to 1,400 ms with reference to stimulus onsets. Extracted epochs were grouped and labeled by trial type (i.e., old, new, and control).

### 2.4 Frequency-specific EEG connectivity analysis

Subsequent memory outcome was extracted using features from the frequency-specific connectivity across multiple memory-related brain areas and memory-related frequency bands. We applied continuous wavelet transformation for the time–frequency analysis. We focused on theta (3–8 Hz) and gamma (30–50 Hz) frequency bands, as they are shown in previous research to correlate with recognition activity. We normalized these power values by the baseline activity before the stimulus onset of −100 to 0 ms. EEG signals after the stimulus onset at 0–500 ms were used as an “test” phase for subsequent connectivity analysis. Considering the time it takes the subject to perceive and recognize the stimuli, the retrieval phase was defined as 500 ms post-stimulus. We include data from regions involved in the memory-related network that were selected previous memory-related studies (i.e., frontal, temporal, parietal, and occipital). Then we used MI, which evaluates the amount of information about one signal that is contained in another signal [19], as our connectivity measure. For the connectivity analysis, we calculated the time-frequency cross-MI [20]. Therefore, following the continuous wavelet transformation, we obtained the mean value of each frequency band (theta and gamma). After cross-MI values were calculated using retrieval samples, we examined the differences in EEG connectivity between correctly recognized and correctly rejected trials.

### 2.5 Subject classification using single-trial connectivity features

**Figure 2:**
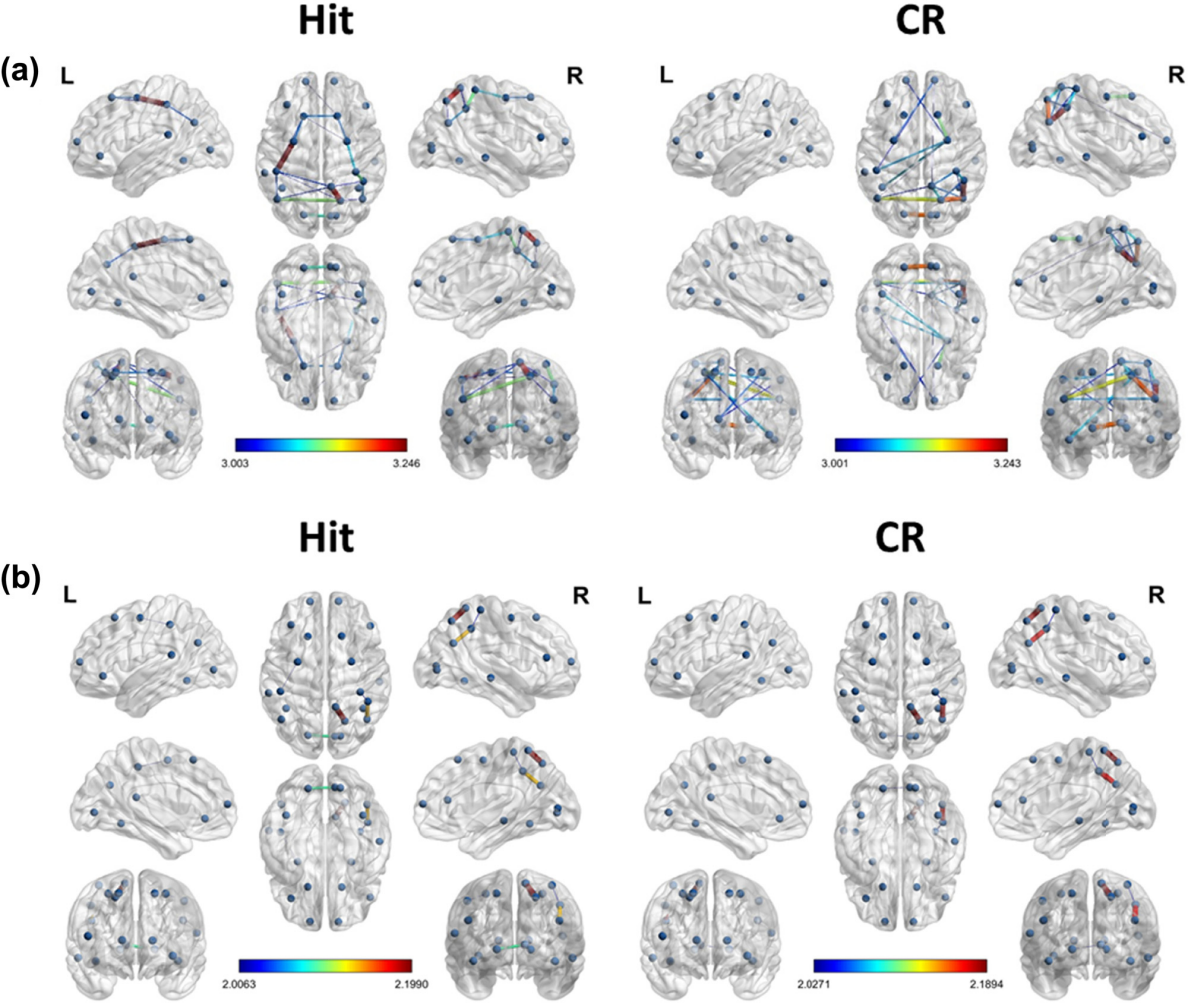
Result of the *t*-test for the brain connectivity of MI (hit vs CR, *p* < 0.05). The nodes selected in this study are PFC, DLPFC, VLPFC, FC, TC, PC, and OC. (a) Theta band brain connectivity of MI during hit (left) and CR (right) conditions. (b) Gamma band brain connectivity of MI during hit (left) and CR (right) conditions.

We used a linear SVM in MATLAB for the classification of memory success. The most informative connectivity values within the top 20% of the *t*-statistics were selected as the features of successful retrieval for each frequency band (Figure 2). The most informative power was selected as a candidate feature for SVM learning to identify the optimal classifier modified from a previous study [21]. SVM group classification analyses were performed using the Statistics Toolbox in Matlab software (version R2018b; MathWorks Inc., Natick, MA, USA). Nonlinear radial basis function kernel (sigma = 2) and constant soft margin (cost = 1) were applied for the SVM training. In the SVM training procedure, the decision boundary formulated using a candidate feature set was optimized to maximize the group classification accuracy. This was achieved by incorporating 80% of trials randomly selected from the total trials [22]. All SVM procedures, testing, and iterative group classifier performance evaluation (with random permutation of subjects into training and testing sets for cross-validation) were repeated 10,000 times per candidate feature set. The classification performance of individual EEG signal was evaluated by five-fold cross-validation with 100 repetitions.

## 3 Results

### 3.1 Behavioral results

On an average, participants correctly remembered 84 ± 0.09% (mean ± standard deviation) of all trials, indicating that they were able to effectively encode materials and that we obtained sufficient trials for both remembered and forgotten conditions (Table 2).

**Table 2:** Average (%) behavioral results of the test session

	Mean	Standard deviation
Accuracy		
Hit	0.73	0.12
CR	0.95	0.06
Miss	0.27	0.12
False alarm	0.05	0.06
Response time (ms)		
Hit	650.21	64.10
CR	579.95	44.75

### 3.2 Frequency-specific EEG feature selection

Table 3 shows the selected features for each frequency band, as well as the *t*-statistics values and regions of the chosen connectivity. The most informative connectivity values within the top 20% of the *t*-statistics were selected as the features for correctly recognized (hit) condition and control trials (CR) for each frequency band. The correctly recognized condition presented bigger theta frequency band connectivity in regions including the left hemispheric fronto-parietal region (i.e., PFC-PC) and right temporo-parietal region during successful recognition phase. On the contrary, correctly rejected new trials showed a significant increase in right hemispheric temporo-occipital connectivity.

**Table 3:** Results of the *t*-test the hit and CR conditions

Hit				CR			
Band	Feature set	*p*-Value	Side	Band	Feature set	*p*-Value	Side
Theta	PFC PC	0.003*	L	Theta	TC OC	0.0084*	R
	TC PC	0.0041*	L		OC OC	0.0090*	LR
	Central OC	0.0091*	R		TC PC	0.0075*	R
Gamma	Central OC	0.0077*	R	Gamma	Central OC	0.0087*	R
	TC OC	0.0085*	R		TC OC	0.0094	R
	OC OC	0.015*	LR				

*p < 0.05, **p < 0.01. PFC, prefrontal cortex; DLPFC, dorsolateral prefrontal cortex; VLPFC, ventrolateral prefrontal cortex; TC, temporal cortex; PC, parietal cortex; OC, occipital cortex, L, left; R, right.

The gamma frequency band also showed increased connectivity in right hemispheric central-occipital regions for both correctly recognized and correctly rejected conditions. On the other hand, a significant change in connectivity was not found in the left hemispheric region in the gamma frequency band for neither condition. Overall, correct recognition was related to the connectivity of the left fronto-parietal network for the theta frequency, whereas the correctly rejected new condition was found to be related to the connectivity of right temporo-occipital regions.

### 3.3 Classification accuracy

Table 4 describes the individual classification accuracy for each frequency band. The SVM binary classifier achieved the highest mean classification accuracy of 86.79 \pm ] 5.93% (mean ± standard deviation) for the theta band.

**Table 4:** Individual subject classification accuracy

Subject	Recognition (0–500 ms) (%)		
	Theta (3–8 Hz)	Gamma (30–50 Hz)	Number of trials (hit/CR)
Sub1	89.09	60.81	48/58
Sub2	89.32	57.63	44/59
Sub3	90.70	51.21	49/59
Sub4	84.23	51.13	47/57
Sub5	90.11	68.25	45/54
Sub6	95.50	59.25	43/59
Sub7	90.68	49.06	42/56
Sub8	85.85	59.79	55/58
Sub9	88.82	53.88	46/60
Sub10	90.59	55.63	46/51
Sub11	85.33	43.88	57/58
Sub12	83.73	42.94	51/60
Sub13	95.86	53.38	55/54
Sub14	78.25	53.25	40/55
Sub15	81.22	50.30	44/60
Sub16	90.45	48.75	40/59
Sub17	73.30	57.07	47/58
Sub18	92.17	50.13	51/60
Sub19	84.41	51.13	55/54
Sub20	81.77	52.06	40/55
Sub21	81.95	50.14	53/59
Sub22	90.27	52.00	55/60
Sub23	86.85	45.75	42/59
Sub24	88.23	50.69	44/60
Sub25	95.44	39.07	47/58
Sub26	77.50	52.38	42/53
Sub27	94.23	51.69	47/59
Sub28	91.91	49.78	47/50
Sub29	75.57	45.80	56/53
Sub30	80.50	43.93	50/56
Average	**86.79**	**51.69**	**47.6/57.03**
SD	**5.93**	**5.85**	**5.10/2.83**

## 4 Discussions

The purpose of this study was to investigate the feasibility of incorporating single-trial connectivity measures as classifiers in predicting one’s future memory performance. Our results revealed that connectivity measures retrieved from time domain EEG data can in fact be used in classifying subsequent memory outcome (i.e., hit vs CR). Furthermore, we were able to predict subsequent memory outcome with the single-trial theta band connectivity feature with a prediction accuracy of 86.79 ± 5.93%. Such results give strong support for the feasibility of single-trial EEG connectivity as a classification feature and achieved a higher classification accuracy than the previous studies that used local brain activities as classifiers (Table 5).

**Table 5:** Talairach coordinates of ROI

Area	Brodmann area	*x*	*y*	*z*
‘O2’	18R	15.445	−80.241	1.209
‘O1’	18L	−17.944	−79.578	1.471
‘Oz’	17R	9.722	−80.832	5.779
‘Pz’	7R	20.152	−64.938	52.17
‘P4’	39R	43.234	−63.01	29.262
‘CP4’	40R	43.95	−44.701	44.334
‘P8’	37R	39.008	−51.835	−10.207
‘C4’	1R	36.017	−34.877	63.832
‘T8’	21R	56.124	−24.4	−6.911
‘P7’	37L	−40.675	−51.795	−9.88
‘P3’	39L	−45.702	−63.239	29.779
‘CPz’	5R	10.565	−50.376	65.651
‘FC4’	6R	26.821	−3.144	55.585
‘C3’	2L	−46.592	−34.283	48.439
‘Fz’	8L	−18.278	22.911	55.919
‘F4’	8R	16.811	23.184	56.088
‘F8’	45R	46.494	33.754	14.35
‘T7’	42L	−58.73	−35.916	17.141
‘FC3’	6L	−28.722	−3.387	55.569
‘FP2’	10R	13.505	59.285	10.292
‘F7’	47L	−35.645	35.003	−4.961
‘FP1’	10L	−16.038	59.012	9.959

Previous studies have explored the role of local brain activities, which is the activation of specific brain regions, linked to recognition in the medial temporal lobe and prefrontal cortex [23–25] and throughout the parietal cortex [26–28]. Recent studies have implied the engagement of the rather widely distributed cortical network and the significance of its integrated roles in the memory retrieval, possibly explaining the improved classification accuracy that we observe in our results. In regard of functional connectivity approaches, increased connectivity in core regions such as prefrontal cortex, precuneus, and occipital cortex is related to successful memory retrieval [17]. King et al. and Jun et al., also exhibited that memory (i.e., recollection)-dependent wide-spread functional connectivity of seed regions within an essential memory network comprised of the angular gyrus, medial prefrontal cortex, hippocampus, middle temporal gyrus, and posterior cingulate cortex is related with behavioral performance [29,30]. These studies illustrate the importance of incorporating the brain activity of multiple regions when classifying memory performance. However, these studies used brain activity in limited areas (e.g., spectral power or ERP of specific regions) as a classification feature.

The present study suggests that subsequent memory outcome prediction accuracy can be improved by using functional connectivity rather than local brain activity, in cases where single-trial classification is performed using scalp EEG data. By using single-trial EEG connectivity features, we were able to achieve a mean accuracy better than above 80% for the classification of subsequent memory outcome. Such improved accuracy could be attributed to the fact that our approach reflects the way in which relevant brain regions communicate during information retrieval.

We found anterior and posterior connectivity of the memory-related network in correctly recognized conditions, in that the connectivity of the left hemispheric fronto-parietal distinctly increased, while that of the right hemispheric network decreased during correctly recognized trials. These results converge with previous fMRI studies that show left-lateralized fronto-parietal brain connectivity for correctly recognized items [26,31,32]. Such left lateralization can be observed in other neural substrates of successful recognition as well. Some attribute such lateralization tendencies of the parietal old/new effect to the verbalization of stimuli. However, the parietal old/new effect was left-lateralized even when facial images were presented instead of words, indicating that the lateralization of the effect was dependent on the region, especially, the parietal cortex [26].

## 5 Conclusion

The present study verified the feasibility of using single-trial functional connectivity in the retrieval phase by using EEG signals for identification of the subsequent memory outcome. We achieved mean accuracy of better than 80% for the prediction of correctly recognized condition. We found the connectivity of the left fronto-parietal cortex higher in correctly recognized condition, whereas that of the left fronto-parietal cortex was not significant in correctly rejected conditions. For future studies, these results could be valuable in building a classification system for memory prediction that could utilize subsequently remembered and forgotten events. Furthermore, we suggest that future work maximize the effectiveness of classification accuracy by adopting computational implementations of models, which include fusion of memory images in wavelet domain and finest architectures of machine learning using a recurrent neural network [33,34].
